# Polyurethane Foam Waste Upcycling into an Efficient and Low Pollutant Gasification Syngas

**DOI:** 10.3390/polym14224938

**Published:** 2022-11-15

**Authors:** Rezgar Hasanzadeh, Parisa Mojaver, Shahram Khalilarya, Taher Azdast, Ata Chitsaz, Mehran Mojaver

**Affiliations:** Department of Mechanical Engineering, Faculty of Engineering, Urmia University, Urmia 5756151818, Iran

**Keywords:** gasification process, plastic waste, waste treatment, polyurethane foam

## Abstract

Waste treatment has attracted much attention and, in this regard, gasification processes offer an efficient thermochemical technique that can produce a syngas rich in hydrogen. This technique has been well developed for solid waste and biomass while investigations on gasification of polymeric foam are rare. Therefore, this study explores the treatment of polyurethane foam waste with different gasifying agents, based on thermodynamic modeling. The polymeric foam gasification was developed using the best model for estimating higher heating value (gross calorific value). As the results indicated, models based on both ultimate and proximate analyses had better performance in predicting higher heating value. As one of the main objectives and novelties, the steam and air gasification performance of flexible and rigid polyurethane foam wastes was investigated and compared from efficiency and CO_2_ emission viewpoints. Polyurethane foam gasification by steam resulted in higher hydrogen efficiency, led to lower energy efficiency and produced lower CO_2_ emissions compared to gasification by air. A hydrogen efficiency of 41.4% was obtained for gasification of waste flexible polyurethane foam by steam. An energy efficiency of 76.6% and CO_2_ emission of 7.43 g per mole of feedstock were attained for waste flexible polyurethane foam gasified by air.

## 1. Introduction

There is a strong demand for progress in developing high-performance and lightweight materials for applications in aerospace, electronics, insulation and automotives [1], leading to the evolution of polymeric foams [2]. Foaming generates a cellular structure in a polymeric matrix [3] which improves its physical and mechanical properties [4] and endows it with excellent sound [5] and thermal insulation performance [6]. Polyurethane (PU), as a special type of polymeric material, has acquired numerous applications as a consequence of its unique properties such as appropriate chemical resistance, good mechanical properties and easy processability [7]. One of the most prevalent PU based materials is PU foam, which is generally categorized into the two categories of rigid and flexible PU foams. Due to the widespread and growing use of PU foams worldwide [8], their waste is increasing.

The issues and concerns involved in waste disposal can be addressed by use of a gasification process [9]; this thermochemical approach has been established for solid waste [10] and biomass [11] and recently developed for waste plastics.

Dang et al. [12] predicted and optimized syngas yield in an air-steam wood residue gasification process and concluded that increasing the temperature, the ratio of steam to biomass and moisture content and decreasing the equivalence ratio enhanced H_2_ yield. AlNouss et al. [13] compared steam and oxygen gasification of a biomass consisting of date pit waste, food waste, manure and dried sludge and concluded that the overall performance of steam gasification was better than oxygen gasification. Policella et al. [14] studied carbon dioxide assisted automobile waste tires gasification and compared its performance with pyrolysis, concluding that the gasification process resulted in higher syngas and lower char formation at the same temperature compared with pyrolysis. Their results indicated that syngas and H_2_ contents were improved with increase of gasification temperature. Mojaver et al. [15] compared the gasification performance of several biomass types by steam considering syngas composition and energy, hydrogen and cold gas efficiencies as criteria and concluded that eucalyptus was the best candidate. AlNouss et al. [16] compared steam gasification performances of coconut coir pith and char and reported that char gasification resulted in a higher syngas output of 1425.92 kg/h compared to 1088.45 kg/h for the pith. Generally, coir pith char gasification caused higher H_2_ and CO production and lower CO_2_ content. Higher H_2_ content was obtained at higher temperatures and higher steam to feedstock ratios.

Research on waste plastic gasification is now underway. For instance, Singh et al. [17] studied and compared the performance of pyrolysis and carbon dioxide assisted gasification of cross-linked polyethylene and concluded that gasification had 2.5 times higher syngas content and higher efficiency compared to pyrolysis. Their results showed that H_2_ production in gasification of cross-linked polyethylene was improved at higher temperatures. Bai et al. investigated supercritical water gasification of polyethylene terephthalate [18] and polypropylene [19] and concluded that its performance was improved at higher temperatures and by increased duration. Jeong et al. [20] studied active carbon assisted polyethylene steam gasification and concluded that H_2_ content was improved significantly by adding active carbon; H_2_ content was about 65 vol%. Steam gasification of waste polyethylene was assessed by Hasanzadeh et al. [21] and the heat required for the gasification process and exergy efficiency were optimized. They reported that increasing the gasification temperature enhanced gasification performance; the optimum conditions to achieve the maximum exergy efficiency and the minimum heat required were at simultaneous low levels of gasification temperatures and steam/waste plastic ratios. Mojaver et al. [22] investigated and compared the steam gasification performance of waste polypropylene, polyethylene, polyethylene terephthalate and polycarbonate and concluded that waste polyethylene gasification was better, considering H_2_ and exergy destruction rate. Four types of plastic waste and six types of conventional biomass were gasified by air and their performance was compared by Mojaver et al. [23]. They concluded that the performance of air gasification of waste plastic was better that for conventional biomass, from the viewpoint of syngas composition and lower heating value.

Although the gasification process of solid waste and biomass has been relatively well established, research on polymeric foam gasification is rare [24]. It is worth noting that the limited numbers of publications [25] in this area are experimental and the theoretical models for gasification of polymeric foams for comprehensive investigation are very limited, to the best of the authors’ knowledge. It should be noted that there are some publications on the optimization of the gasification process of polymeric foams and they are based on experimental findings [26]. It is important to note also that a comprehensive comparison between gasification of flexible and rigid polyurethane foam wastes is not fully reported. Hence, the main novelties and contributions of the present research work can be highlighted as: comparative analysis on performance assessment of gasification of flexible and rigid polyurethane foam waste with respect to energy efficiency, hydrogen efficiency and CO_2_ emission; presenting theoretical models for assessing the gasification process of polymeric foams considering flexible and rigid polyurethane foam waste as a case study; comparing different models to calculate higher heating values of polymeric foams and assessing their performance to estimate this value with the minimum error; evaluating the effects of key characteristics including temperature, feedstock moisture content, steam/waste foam and equivalence ratios on syngas composition, hydrogen efficiency, and energy efficiency; comparing the performance of air- and steam-gasification of flexible and rigid polyurethane foam wastes.

## 2. Gasification Modeling

**Figure 1:**
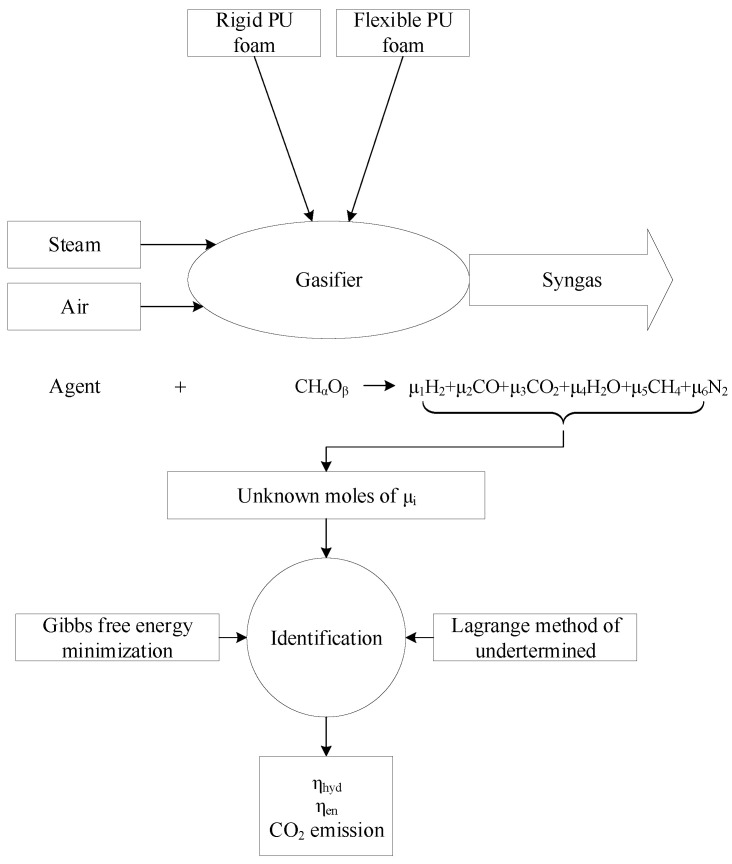
Schematic of the modeling procedure.

The air and the steam gasification processes of flexible and rigid PU foam waste are modeled in this study based on a thermodynamic model using a coupling method of Lagrange of undetermined multipliers/Gibbs free energy minimization. Waste flexible or rigid PU foam enters the gasifier reactor as the feedstock option and air or steam enter the reactor as the gasifying agent option. The molar composition of the syngas is unknown and is identified based on the coupling method utilized in this study. After identification of the molar composition, the hydrogen and energy efficiencies and CO_2_ emission are calculated as the system performance indicators. The schematic of the modeling procedure in this study is presented as a block diagram in Figure 1.

Feedstock gasification results in a combustible syngas consisting H_2_, CO, CO_2_, CH_4_ and N_2_ (in the case of air gasification) in the presence of an oxygen carrier as gasifying agent. Steam and air are among the most relevant gasifying agents: steam gasification results in a significant hydrogen production while air gasification lead to lower CO_2_ emission and has lower cost. Hence, both steam and air gasifying agents were considered for this study and their performance was compared in detail. As one of the main objectives of the present research work, waste flexible and rigid PU foams were selected as the feedstock types and their proficiencies were investigated in the gasification process. The feedstock is reacted in the gasifier reactor with the gasifying agent, comprising evaporated water after passing through a heat exchanger in the steam case and air passed through an air conditioner in air gasification. Steam and air gasification of waste flexible and rigid PU foams was assessed per unit feedstock mole in the present study. Equations (1) and (2) indicate the global reactions of steam and air gasification processes, respectively [15]:

(1) $${\mathrm{CH}}_{\alpha }O_{\beta }+\left(\gamma +\delta \right)H_{2}O\to \mu_{1}H_{2}+\mu_{2}\;\mathrm{CO}+\mu_{3}{\mathrm{CO}}_{2}+\mu_{4}H_{2}O+\mu_{5}{\mathrm{CH}}_{4}$$

(2) $${\mathrm{CH}}_{\alpha }O_{\beta }+\varepsilon \left(O_{2}+3.76N_{2}\right)\to \mu_{1}H_{2}+\mu_{2}\mathrm{CO}+\mu_{3}{\mathrm{CO}}_{2}+\mu_{4}H_{2}O+\mu_{5}{\mathrm{CH}}_{4}+\mu_{6}N_{2}$$

where α denotes the molar ratio of hydrogen to carbon and β indicates the molar ratio of oxygen to carbon in the feedstock chemical formula, respectively. γ and δ are moisture contents of feedstock and feeding steam, respectively, and ε is feeding air. $\mu_{i}$ are moles number of products which are H_2_, CO, CO_2_, H_2_O, CH_4_ and N_2_ (in air gasification) and are unknown values which should be identified.

This study utilizes a coupling method of Lagrange of undetermined multipliers/Gibbs free energy minimization which has been presented in [27]. This technique has several advantages such as independence from defining the reactions and convergence of the computations and it does not require the estimation of the initial equilibrium compositions.

The gasification processes of waste flexible and rigid PU foams were evaluated using hydrogen ($\eta_{hyd}$) and energy ($\eta_{en}$) efficiencies obtained as [28]:

(3) $$\eta_{hyd}\;\left(\%\right)=\frac{\lambda_{\mathrm{hydrogen}}}{E_{in}}$$

(4) $$\eta_{en}\;\left(\%\right)=\frac{\lambda_{\mathrm{hydrogen}}+\lambda_{\mathrm{carbon}\;\mathrm{monoxide}}+\lambda_{\mathrm{methane}}}{E_{in}}$$

where $\lambda_{i}$ is energy of the *i*th component and $E_{in}$ is input energy.

$\lambda_{i}$ for each component is as:

(5) $$\lambda_{i}=\mu_{i}\times \sigma_{i}$$

where $\sigma_{i}$ is lower heating value of the *i*th component.

Proximate and ultimate analyses of waste flexible [29] and rigid [24] PU foams are extracted from the literature.

## 3. Results and Discussion

### 3.1. Calculation of Higher Heating Value

The modeling utilized in this study needs the higher heating values for gasification performance evaluation. On the other hand, Guo et al. [26] and Wu et al. [29] do not provide these values for rigid and flexible PU foams. Hence, different models are used to estimate the higher heating values. Firstly, the accuracies of these models are checked using experimental data available in the literature for waste polystyrene foam, and then, higher heating values are calculated using the best model.

There are many models presented for higher heating value calculation, most of which have been reported in Vargas-Moreno et al. [30]. These models are generally based on ultimate analysis and/or proximate analysis. From each category, the most recent, prevalent and applicable ones were selected.

Friedl et al. [31] proposed the following equation for higher heating value calculating based on ultimate analysis:

(6) $$\xi_{i}=-2.230\;H+0.0512\;\left(C\times H\right)+0.00355\;C^{2}-0.232\;C+0.131\;N+20.600$$

The following equation was proposed by Sheng and Azevedo [32] for higher heating value calculating:

(7) $$\xi_{i}=+0.7009\;H+0.3137\;C+0.0318\;\left(O+N\right)-1.3675$$

Thipkhunthod et al. [33] presented higher heating value as follows:

(8) $$\xi_{i}=-0.0698\;H+0.4259\;C-0.1805\;N+0.1817\;O-2.2770$$

Higher heating value was calculated based on carbon and hydrogen in ultimate analysis by Yin [34] as follows:

(9) $$\xi_{i}=0.2949\;C+0.8250\;H$$

Callejón-Ferre et al. [35,36] presented the following equation for calculating higher heating value based on ultimate analysis:

(10) $$\xi_{i}=-0.433\left(H+N\right)+0.517\;\left(C+N\right)-3.440$$

Sheng and Azevedo [32] presented the following equation for calculating higher heating value based on proximate analysis:

(11) $$\xi_{i}=19.914-0.2324\;\mathrm{Ash}$$

Huang et al. [37] proposed this equation:

(12) $$\xi_{i}=18.96016-0.22527\;\mathrm{Ash}$$

Callejón-Ferre et al. [35,36] presented the following equation to obtain higher heating value based on proximate analysis:

(13) $$\xi_{i}=20.086-0.261\;\mathrm{Ash}$$

As mentioned previously, several models are based on proximate and ultimate analyses to estimate higher heating value. Grabosky and Bain [38] presented the following equation:

(14) $$\xi_{i}=1.4306\;H+0.328\;C-0.0237\;N+0.0929\;S-\left[\left(\frac{40.11\;H}{C}\right)\times \left(1-\frac{\mathrm{Ash}}{100}\right)\right]$$

Channiwala and Parikh [39] proposed the following equation:

(15) $$\xi_{i}=1.1783\;H+0.3491\;C-0.0151\;N+0.1005\;S-0.1034\;O-0.0211\;\mathrm{Ash}$$

Thipkhunthod et al. [33] presented two models for higher heating value as follows:

(16) $$\xi_{i}=-0.2106\;H+0.4064\;C-0.1513\;N+0.1547\;S+0.1603\;O-0.0238\;\mathrm{Ash}+0.0034$$

(17) $$\xi_{i}=0.9703\;H+0.2243\;C-0.0000238\;N+0.0000928\;S+0.1546\;O-0.0331\;\mathrm{Ash}$$

The following equations were presented by Callejón-Ferre et al. [35,36]:

(18) $$\xi_{i}=-0.385\;H+0.475\;C+0.102\;N-0.0251\;\mathrm{Ash}-1.563$$

(19) $$\xi_{i}=-0.376\;H+0.475\;C+0.099\;N-0.024\;\mathrm{Ash}-1.642$$

To check the accuracies of these models, the predicted higher heating values are compared with the experimental data available in [40,41] for waste polystyrene foam. The ultimate analysis of waste polystyrene foam was reported as 91.17% of C, 8.33% of H, 0.18% of O, 0.32% of N and 0% of S and its proximate analysis was obtained as 0% of moisture, 99.59% of combustible and 0.06% of ash [40,41]. The higher heating values estimated by the presented models are tabulated in Table 1 along with their errors compared to the experimental data of [40,41].

The results indicated that the models based on ultimate analysis, i.e., Equations (6)–(10), had acceptable errors in estimating the higher heating value of polystyrene foam, albeit the accuracy of the model presented by Callejón-Ferre et al. [35,36], i.e., Equation (10) was considered appropriate with an error of only 3.24%. According to the results shown in Table 1, the models proposed based on proximate analysis, i.e., Equations (11)–(13), did not make good estimates, since all of them underestimated higher heating values with errors more than 50%. The findings in Table 1 revealed that models based on both proximate and ultimate analyses are the best models to predict the higher heating values of polymeric foams and their accuracies are appropriate. It is noteworthy that the error of the model proposed by Channiwala and Parikh [39], i.e., Equation (15), was only 0.38% and its validity was confirmed for estimating the higher heating value of polymeric foams. Therefore, this model was utilized for estimation of the higher heating values of waste flexible and rigid PU foams.

Proximate and ultimate data of waste flexible [29] and rigid [26] PU foams are replaced in Equation (15). The higher heating value of waste rigid PU foam was found to be 33.18 MJ/kg and the value for flexible PU foam waste was obtained as 32.45 MJ/kg.

### 3.2. Model Validation

The syngas composition of both steam and air gasification was compared with the results available in the literature to validate the accuracies of the model used in this study. The results are presented in Table 2. For validation of steam gasification, experimental results reported in Wu and Williams [42] and theoretical data proposed in Saebea et al. [43] were compared with the results obtained by the model presented in this study. The feedstock was waste polypropylene. The gasification was accomplished at gasification temperature and pressure of 800 °C and 101.3 kPa, respectively. The findings revealed agreement between both experimental and theoretical data showing the validity of steam gasification.

For validation of air gasification, the results were compared with those reported in Cho et al. [44]. The feedstock was a mixture of waste plastic. The gasification was accomplished at gasification temperature and pressure of 803 °C and 101.3 kPa, respectively. The results in Table 2 show agreement with the results reported in Cho et al. [44]. Hence, the model of air gasification was also verified.

### 3.3. Gasification Assessments

**Figure 2:**
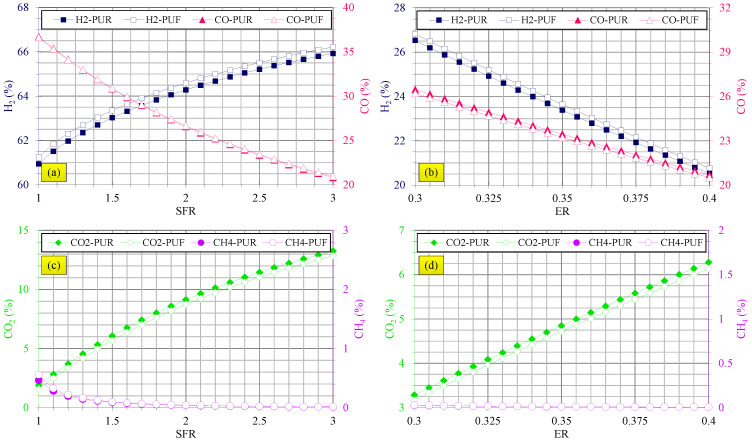
Effects of SFR and ER on syngas composition. (**a**) Effects of steam/waste PU foam ratio on H_2_ and CO yields in steam gasification, (**b**) effects of equivalence ratio on H_2_ and CO yields in air gasification, (**c**) effects of steam/waste PU foam ratio on CO_2_ and CH_4_ yields in steam gasification and (**d**) effects of equivalence ratio on CO_2_ and CH_4_ yields in air gasification.

Figure 2 shows the effects of the steam/waste PU foam ratio in gasification by steam and equivalence ratio in air gasification on syngas composition.

Figure 2a indicates that H_2_ content was improved and CO yield was reduced at higher steam/waste PU foam ratios. Figure 2c shows that CO_2_ content was increased and CH_4_ yield was slightly mitigated with steam/waste PU foam ratio especially at low SFRs while total CH_4_ yield was almost negligible and was less than 1%. It is noteworthy that these trends were similar for both waste flexible and rigid PU foams. These observations can be justified by the following explanations. Higher steam/waste PU foam ratio means higher steam fed to the gasifier; therefore, more H_2_O is available for the water-gas shift reaction, presented in Equation (20) and steam reforming reaction, presented in Equation (21) [45]:

(20) $$\mathrm{CO}+H_{2}O↔{\mathrm{CO}}_{2}+H_{2}$$

(21) $${\mathrm{CH}}_{4}+H_{2}O↔\mathrm{CO}+3H_{2}$$

Both reactions are shifted to the product side at higher steam/waste PU foam ratio and, therefore, H_2_ and CO_2_ yields increase and CO content is consumed according to the water-gas shift reaction, consistent with the results of Figure 2a,c. According to the steam reforming reaction, CH_4_ is expended and decreases at higher H_2_O content and, therefore, the CH_4_ decrement shown in Figure 2c is logical.

It should be noted that based on the results shown in Figure 2a,c, waste flexible PU foam produced higher H_2_ and lower CO_2_ yields in comparison to waste rigid PU foam at all steam/waste PU foam ratios. Their CO and CH_4_ yields were comparable. Comparison of these results with the literature for other feedstock types, such as biomass and plastic waste, confirms the authenticity of the results. For instance, Marcantonio et al. [46] assessed gasification of hazelnut shells and showed that increasing the ratio of steam to hazelnut shells increased H_2_ and CO_2_ yields and decreased CO and CH_4_ yields. These observations are in agreement with the results of the present study. Li et al. [47] studied steam gasification of three types of biomass and concluded that a higher steam to biomass ratio augmented H_2_ and CO_2_ yields and reduced CO yield, while the CH_4_ yield was negligible. Their findings agree with the results presented in this study. In steam gasification of wood residue [48], biomass char [49], pine sawdust [50] and wood pellets [51], similar trends were described. For plastic waste gasification [21,22], similar tendencies were also observed.

Figure 2b,d demonstrate that H_2_ and CO were lessened and CO_2_ was increased at higher equivalence ratios in air gasification of both flexible and rigid PU foam waste. The CH_4_ yield was negligible in air gasification of flexible and rigid PU foam waste at all equivalence ratios. These observations are justified as follows.

Hydrogen and carbon monoxide oxidations are as follows, respectively [52]:

(22) $$H_{2}+0.5O_{2}\to H_{2}O$$

(23) $$\mathrm{CO}+0.5O_{2}\to {\mathrm{CO}}_{2}$$

Higher CO_2_ is available at higher equivalence ratios due to higher O_2_ content for carbon monoxide and hydrogen oxidation processes. Hence, these reactions are more activated at high levels of equivalence ratio. Hence, more H_2_ and CO are consumed and their contents are decreased while more CO_2_ is produced.

For air gasification of waste plastics [23], distiller grains with solubles [53], rice husk gasification [54], solid refuse fuel [55], wood pellets [56] and eucalyptus chips [57], similar findings were conveyed.

The findings reveal that air gasification of waste flexible PU foams produced more H_2_ yield and less CO and CO_2_ yields compared with waste rigid PU foams, as the results of Figure 2b,d reveal.

**Figure 3:**
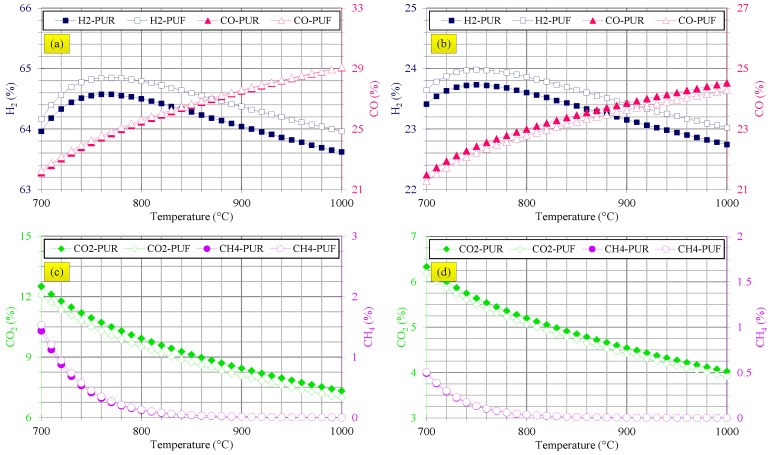
Effects of gasification temperature of waste flexible and rigid PU foams on (**a**) H_2_ and CO yields in steam gasification, (**b**) H_2_ and CO yields in air gasification, (**c**) CO_2_ and CH_4_ yields in steam gasification and (**d**) CO_2_ and CH_4_ yields in air gasification.

The influence of temperature on performance of steam and air waste flexible and rigid PU foams gasification is depicted in Figure 3. According to the results of Figure 3a, H_2_ yield was firstly enhanced and, then, was mitigated at higher temperature in steam gasification while CO yield was continuously increased. A decreasing trend was observed in CO_2_ and CH_4_ yields versus temperature in steam gasification, as the results of Figure 3c show. All of these trends were similar for both waste flexible and rigid PU foams. These findings are justified based on the water-gas shift (Equation (20)), water-gas (Equation (24)) and Boudouard (Equation (25)) reactions [45]:

(24) $$C+H_{2}O\to \mathrm{CO}+H_{2}$$

(25) $$C+{\mathrm{CO}}_{2}\to 2\mathrm{CO}$$

Both water-gas and Boudouard are endothermic reactions and, therefore, they are activated at higher temperatures. Based on the water-gas reaction, more H_2_ and CO are produced, and based on the Boudouard reaction, more CO_2_ is consumed and CO is produced at higher gasification temperatures. Therefore, the changes observed in the syngas compositions as depicted in Figure 3a,c are confirmed. The declining trend of H_2_ at high temperatures is explained by the water-gas shift reaction. It is an exothermic reaction and increasing temperature shifts this reaction to the left side and, therefore, consuming H_2_ led to this declining trend. Cracking CH_4_ to other species at higher temperatures can be a possible reason for its decline. These phenomena are in accordance with the findings reported for biomass stalk [47], sewage sludge [58], waste polyethylene [43], municipal solid waste [59], different waste plastics [22], wood, coconut shell and straw [60] and waste polyethylene [21].

The findings depicted in Figure 3a,c reveal that steam gasification of flexible PU foam resulted in higher H_2_ yield and lower CO_2_ yield at all gasification temperatures and their CO and CH_4_ yields were almost the same.

Figure 3b,d show that the effects of temperature in air gasification of waste flexible and rigid PU foams are similar to those for steam gasification. H_2_ yield was firstly improved with increasing gasification temperature and then experienced a decline. CO yield was increased and CO_2_ and CH_4_ yields were reduced with temperature. These behaviors applied to both waste flexible and rigid PU foams. It should be notified that air gasification of flexible PU foams yielded higher H_2_ and lower CO and CO_2_ contents at all gasification temperatures compared with rigid PU foams. The explanations about the changes of syngas composition versus temperature in air gasification of waste flexible and rigid PU foams are similar to those previously presented for steam gasification. Similar observations were made for gasification of solid refuse fuel by air [55], co-gasification of digestate and lignite [61] and waste plastics [29].

**Figure 4:**
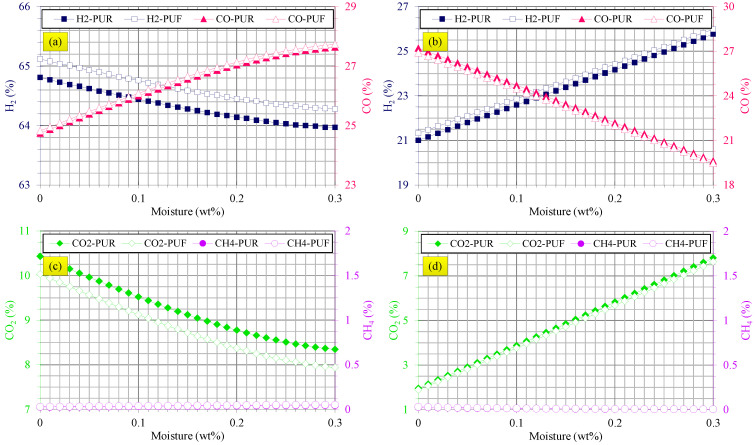
Effects of moisture content of waste flexible and rigid PU foams on (**a**) H_2_ and CO yields in steam gasification, (**b**) H_2_ and CO yields in air gasification, (**c**) CO_2_ and CH_4_ yields in steam gasification and (**d**) CO_2_ and CH_4_ yields in air gasification.

Figure 4 shows syngas composition variations versus moisture content. Figure 4a indicates that the H_2_ yield was reduced and the CO yield was increased at higher moisture content in steam gasification of both waste flexible and rigid PU foams.

According to the results shown in Figure 4c, at higher moisture content, the CO_2_ yield of steam gasification was decreased and this phenomenon was similar for waste flexible and rigid PU foams. CH_4_ yield was negligible for steam gasification of waste flexible and rigid PU foams, as the results of Figure 4c reveal. These results agreed with those reported for waste polycarbonate, polyethylene terephthalate, polypropylene and polyethylene [22]. It is worth noting that gasification of waste flexible PU foams by steam produced higher H_2_ yield and lower CO_2_ yield compared with waste rigid PU foams at all moisture contents.

Figure 4b,d illustrate that H_2_ yield was enhanced, CO yield was mitigated and CO_2_ yield was increased at higher moisture contents of both waste flexible and rigid PU foams in air gasification. These trends can be explained by the water-gas shift reaction as in Equation (20). More H_2_O content is available for shifting the water-gas shift reaction to the products at higher moisture levels and, hence, more CO is consumed and more CO_2_ and H_2_ are produced. Hence, increased H_2_ and CO_2_ contents and decreased CO yield at higher moisture contents of waste flexible and rigid PU foams were confirmed.

Similar results were observed for gasification of sewage sludge by air [62] and for municipal solid waste [63]. It should be noted that the differences between the syngas composition of waste flexible and rigid PU foams were negligible at all moisture contents in gasification by air; however, waste flexible PU foams produced slightly higher H_2_ content and lower CO_2_ content in steam gasification.

**Figure 5:**
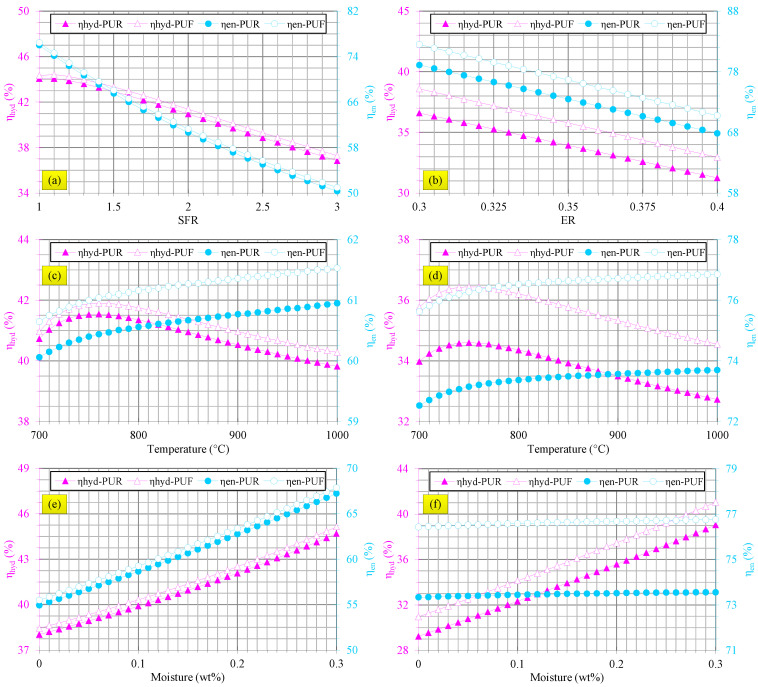
Effects of gasification parameters on energy and hydrogen efficiencies. (**a**) Effects of moisture content of waste flexible and rigid PU foams in steam gasification, (**b**) effects of moisture content of waste flexible and rigid PU foams in air gasification, (**c**) effects of temperature in steam gasification, (**d**) effects of temperature in air gasification, (**e**) effects of steam/waste PU foam ratio in steam gasification and (**f**) effects of equivalence ratio in air gasification.

Figure 5 shows the effects of steam and air gasification parameters on energy and hydrogen efficiencies of waste flexible and rigid PU foams.

Figure 5a reveals that, in steam gasification, both energy and hydrogen efficiencies of waste flexible and rigid PU foams were mitigated at higher steam/waste PU foam ratios. Although the H_2_ yield was increased with the steam/waste PU foam ratio (see Figure 2a), more heat is required for the gasification process and more energy is fed to the gasifier at higher steam/waste PU foam ratios and, therefore, energy and hydrogen efficiencies were reduced. Reduction of CO yield (see Figure 2a) can be another reason for declining energy efficiencies at a higher steam/waste PU foam ratio in gasification by steam. The results demonstrated that energy and hydrogen efficiencies in gasification of waste flexible and rigid PU foams by steam were almost equal. Figure 5b shows that the energy and hydrogen efficiencies of waste flexible and rigid PU foams were decreased at higher equivalence ratio in air gasification process because of decreasing H_2_ and CO yields (see Figure 2b). It is important to note that gasification of waste flexible PU foams by air brought forth higher energy and hydrogen efficiencies at all equivalence ratios compared with those of waste rigid PU foams.

Figure 5c,d display that hydrogen efficiencies in gasification by air and steam were first improved and then lessened at higher gasification temperatures for both waste flexible and rigid PU foams. These trends followed the H_2_ yield versus gasification temperature relationship (see Figure 3a,b). According to Figure 5c,d, energy efficiencies of waste flexible and rigid PU foams were promoted at higher temperatures in air and steam gasification, which was mainly due to rising CO yield with gasification temperature (see Figure 3a,b). It should be pointed out that waste flexible PU foams gasification caused higher energy and hydrogen efficiencies as compared with waste rigid PU foams especially in air gasification.

Figure 5e indicates that both energy and hydrogen efficiencies were improved at higher moisture contents of waste flexible and rigid PU foams in steam gasification while both efficiencies were slightly higher in the case of waste flexible PU foams. Although the H_2_ yield was slightly decreased at higher moisture content, the CO yield experienced an increasing trend (see Figure 4a) and, therefore, efficiencies were boosted with the increase of moisture content. Figure 5f shows that hydrogen efficiency was improved at higher moisture content in air gasification as depicted in Figure 4b. Figure 5f indicates that moisture content did not considerably affect the energy efficiency in air gasification because H_2_ yield was improved and neutralized the reduction of CO yield.

The gasification performances of waste flexible and rigid PU foams with steam and air were compared at the same gasification conditions of 850 °C, steam/waste PU foam ratio of 2, and moisture content of 15% in steam gasification, and 850 °C, equivalence ratio of 0.35, and moisture content of 15% in air gasification. The results are presented in Table 3. Energy efficiency, hydrogen efficiency and CO_2_ emission were criteria for this comparison. The results showed that gasification of waste flexible PU foams was better in comparison to waste rigid PU foams with respect to the considered efficiencies and carbon dioxide emissions in both cases of steam and air gasification. Hydrogen efficiency in the case of steam gasification was higher compared to gasification by air while the latter resulted in higher energy efficiency for both waste types. Air gasification of waste PU foams caused lower CO_2_ emission compared with steam gasification. Steam gasification of waste flexible PU foams had the best performance with respect to hydrogen efficiency with a value of 41.37%. The best performance from the energy efficiency and CO_2_ emission viewpoints was from gasification of waste flexible PU foam by air, with an energy efficiency of 76.64% and CO_2_ emission of 7.43 g.

## 4. Conclusions

Steam and air gasification of waste flexible and rigid PU foams was investigated and compared in detail with respect to the steam/waste PU foam ratio, equivalence ratio, moisture content of waste PU foam, and gasification temperature, considering syngas composition, energy and hydrogen efficiencies and CO_2_ emissions. The main findings and results of this study can be concluded as follows:

* Models based on ultimate and proximate analyses have the best accuracy to predict the higher heating value of polymeric foams; the best one had an error smaller than 1%.

* The H_2_ content of waste PU foam gasification was improved at higher steam/waste PU foam ratios. This improvement was from 60.94% to 65.93% for rigid PU foam and from 61.21% to 66.22% for flexible PU foam, with an increase in the steam/waste PU foam ratio from 1 to 3.

* The H_2_ yield from waste PU foam gasification was decreased at higher equivalence ratios. This decrement was from 26.53% to 20.52% for rigid PU foam and from 26.82% to 20.76% for flexible PU foam, with an increase in equivalence ratio from 0.3 to 0.4.

* There was an optimum temperature to achieve the highest H_2_ content for waste PU foam gasification and at this optimum condition, the flexible PU foam provided 64.85% H_2_ content in steam gasification and 23.98% of H_2_ content in air gasification.

* The H_2_ content of waste PU foam gasification was reduced by increasing the moisture content of the feedstock in the steam gasification. This reduction was from 65.12% to 64.28% for flexible PU foam, with an increase in the moisture content from 0 to 30 wt%.

* H_2_ content of waste PU foam gasification was improved in the air gasification by increasing the moisture content from 0 to 30 wt%. This improvement was from 21.33% to 25.99% for flexible PU foam.

* Flexible PU foam waste gasification resulted in higher energy and hydrogen efficiencies at all processing conditions compared with rigid PU foam waste gasification. For instance, energy efficiency in flexible PU foam waste gasification was 76.76% compared to 73.56% in rigid PU foam waste gasification at a moisture content of 30 wt%.

* Steam gasification of waste PU foam led to higher hydrogen efficiency compared with air gasification. For instance, the hydrogen efficiency of steam gasification of flexible PU foam waste was 41.9% compared to 36.44% for air gasification of flexible PU foam waste at their optimum temperatures.

* Energy efficiency was more and CO_2_ emission was less in air gasification of waste PU foam than its steam gasification.

## Figures and Tables

**Table 1:** Accuracy of models to predict higher heating value of waste polystyrene foam.

Model	$\xi_{i}\;(\mathrm{MJ}/\mathrm{kg})$		Error (%)
	Experimental [40,41]	Theoretical	
Equation (6) [31]	41.46	49.306	18.92
Equation (7) [32]	41.46	33.087	20.20
Equation (8) [33]	41.46	35.946	13.30
Equation (9) [34]	41.46	33.758	18.58
Equation (10) [35,36]	41.46	40.115	3.24
Equation (11) [32]	41.46	19.900	52.00
Equation (12) [37]	41.46	18.947	54.30
Equation (13) [35,36]	41.46	20.070	51.59
Equation (14) [38]	41.46	38.151	7.98
Equation (15) [39]	41.46	41.618	0.38
Equation (16) [33]	41.46	35.280	14.91
Equation (17) [33]	41.46	28.557	31.12
Equation (18) [35,36]	41.46	38.567	6.98
Equation (19) [35,36]	41.46	38.562	6.99

**Table 2:** Comparing the syngas composition for steam and air gasification.

Component	Steam Gasification			Air Gasification	
	Exp. [42]	Model [43]	This Model	Exp. [44]	This Model
H_2_	64	67.25	67.19	18.61	16.59
CO	25.7	25.24	26.51	12.11	13.99
CO_2_	6.4	7.33	6.027	8.88	7.269
CH_4_	3.3	0.18	0.2731	2.24	0.006
N_2_	-	-	-	58.17	62.14

**Table 3:** Performance comparison of steam and air gasification of waste flexible and rigid PU foams.

Output	Steam Gasification		Air Gasification	
	Waste Rigid PU Foam	Waste Flexible PU Foam	Waste Rigid PU Foam	Waste Flexible PU Foam
$\eta_{\mathrm{hyd}}$ (%)	40.95	41.37	33.93	35.78
$\eta_{\mathrm{en}}$ (%)	60.67	61.27	73.49	76.64
CO_2_ emission (g)	11.24	10.84	7.53	7.43

## Data Availability

Not applicable.

## Nomenclature

$E_{in}$	Input energy (J)
$n_{i}$	Molar numbers of products
$\eta_{hyd}$	Hydrogen efficiency (%)
$\eta_{en}$	Energy efficiency (%)
$\lambda_{i}$	Energy of *i*th component (J)
$\sigma_{i}$	Lower heating value of *i*th component (J/g)
C	Carbon
H	Hydrogen
N	Nitrogen
O	Oxygen
*T*	Temperature (K)
α	Hydrogen/carbon molar ratio
β	Oxygen/carbon molar ratio
γ	Moisture content of feedstock (mol)
δ	Feeding steam as the gasifying medium (mol)
ε	Air content per mole of feedstock (mol)
